# Does Electronic Cigarette Use Predict Abstinence from Conventional Cigarettes among Smokers in Hong Kong?

**DOI:** 10.3390/ijerph15030400

**Published:** 2018-02-26

**Authors:** Socrates Yong-da Wu, Man Ping Wang, William H. Li, Antonio C. Kwong, Vienna W. Lai, Tai Hing Lam

**Affiliations:** 1 School of Nursing, University of Hong Kong, Hong Kong, China; yongdang@connect.hku.hk (S.Y.-d.W.); william3@hku.hk (W.H.L.); 2 Hong Kong Council on Smoking and Health, Hong Kong, China; info@cosh.org.hk (A.C.K.); ed@cosh.org.hk (V.W.L.); 3 School of Public Health, University of Hong Kong, Hong Kong, China; hrmrlth@hku.hk

**Keywords:** electronic cigarettes, smoking cessation, nicotine, conventional cigarettes

## Abstract

Objectives: To investigate the effects of ever use of electronic cigarettes (ECs), many of which lack nicotine, on abstinence from convention cigarettes among Hong Kong adult smokers. Methods: We collected data from 956 daily smokers in 2014–2015 regarding ever EC use and smoking behaviors at baseline, any and past 30-day EC use at the 3-month follow-up. Outcomes measured at 6 months included past 7-day point prevalence abstinence (PPA), biochemically validated quitting, smoking reduction (≥50% from baseline) and cessation attempt. Logistic regression yielded adjusted odds ratios (AOR) for quitting in relation to EC use, adjusting for socio-demographic characteristics and smoking profile. Complete case, missing observation as smoking and propensity score analyses were conducted. Results: By complete case, ever EC use at baseline did not predict self-reported PPA (AOR 0.99, 95% CI 0.57–1.73), biochemically validated quitting (AOR 1.22, 95% CI 0.64–2.34), cessation attempt (AOR 0.74, 95% CI 0.48–1.14), or smoking reduction (AOR 0.89, 95% CI 0.54–1.47). EC use during the first 3 months did not predict quitting (AOR 1.02, 95% CI 0.22–4.71). Similar results were observed for missing observations as smoking and propensity score analyses. Conclusions: Any use of ECs, many of which lack nicotine, did not predict smoking abstinence among Hong Kong adult smokers.

## 1. Introduction

Electronic cigarette (EC) use is increasingly popular in Western countries with 5.5% of the adult population in England using EC in the first quarter of 2015 [1] and 3.7% US adults using EC daily or some days in 2014 [2]. Less was known about the prevalence in Asian countries except in Korea, where 6.6% of adults have ever tried EC and 1.1% used EC in the past 30 days in 2015 [3]. Current conventional cigarette users (referred to as smokers hereafter) have higher use prevalence [1] with one of the common purposes being to quit smoking conventional cigarettes [4]. Smokers perceived EC as a potential new smoking cessation aid [5], which might have come from the cessation claims in promotions of EC [6] and the appealing functions of EC in mimicking conventional cigarette’s appearance and usage. Smokers who used EC were also more likely to have a higher level of nicotine dependence and to make a cessation attempt than smokers without EC use [7], suggesting smokers who had failed to abstain from conventional cigarettes because of severe addiction [8] might be tempted to try EC.

EC’s effect on abstinence from conventional cigarettes is controversial and all the longitudinal studies are based on Western populations. Results from meta-analysis studies are inconsistent, with some arguing that nicotine EC were more effective than nicotine-free products for abstinence from conventional cigarettes [9,10], while others found inconclusive [11,12] or negative evidence [13]. A recent meta-analysis including 20 studies with control groups (15 cohort studies, 3 cross-sectional ones, and 2 trials) suggested that EC users were less likely to abstain from conventional cigarettes [13], but studies with different designs reported inconsistent results. Randomized controlled trials (RCTs) tended to show EC’s efficacy, such as reducing craving and withdrawal symptoms [14] and boosting cessation as effective as the nicotine patch [15], under a commonly more intensive use setting. Non-trial longitudinal studies revealed inconsistent results (some reported higher odds of cessations among those who used EC daily or used tank systems [16,17] but others found no or negative associations [18,19]), which were probably due to variations in study settings and the types of EC products, intensity, and method of use [20,21]. Discrepancies in EC’s cessation efficacy between trial and real-world studies, which were similar to those observed for nicotine replacement therapy [22], suggest that EC may not help smokers quit even if it is effect was proven.

In Hong Kong (HK), which has the one of the lowest smoking prevalence (10.7% in 2015) in the developed world [23,24], EC use remains rare (0.7% adult population have ever tried EC and 0.2% used EC in the past 30 days) [25], which may be partly due to its use (defined as smoking by law) being forbidden in smoke-free areas under the comprehensive smoke-free legislation as from 2007 and only ECs without nicotine are widely available under regulations on nicotine. ECs containing nicotine are required to register according to the Pharmacy and Poisons Ordinance (none has done so as of November 2017) and therefore most ECs in HK, without passing any mandatory inspection, are allegedly nicotine-free and can be promoted freely and sold in retail shops and online. Non-nicotine EC was reportedly used by 46.3% EC users in a representative local adult population sample [25], which was much higher than that reported by European EC users (24.2%) [4]. Studies investigating the effect of using EC without nicotine on abstinence from conventional cigarettes are scarce, with only 4 RCTs which compared non-nicotine EC with nicotine products [15,26,27,28]. Two RCTs showed EC without nicotine had weaker effects on smoking abstinence or reduction [26,27], but another 2 RCTs found EC with or without nicotine had a similar effect on quitting or lowering consumption of conventional cigarettes [15,28]. To our knowledge, no published longitudinal observational study on EC and abstinence from conventional cigarettes involved a significant proportion of non-nicotine products. We prospectively studied whether ever EC (irrespective of nicotine strength) use predicted abstinence from conventional cigarettes and other smoking-related outcomes in adult daily smokers in Hong Kong.

## 2. Materials and Methods

### 2.1. Design

We analyzed the data from the 5th “Quit to Win” contest (QTW), which was organized by the Hong Kong Council on Smoking and Health (COSH) and School of Nursing at the University of Hong Kong between June 2014 and March 2015. Details of the QTW have been published elsewhere [29]. In brief, the QTW incorporates incentives, competition, and social support to promote abstinence from conventional cigarettes among adult smokers recruited in the community. Smokers who were residents of Hong Kong, aged 18 or above, able to communicate in Cantonese, and not involved in other smoking cessation programs were recruited (n = 1307) from June to September 2014. Smokers consuming at least 1 cigarette daily in the past 3 months with exhaled carbon monoxide (CO) ≥ 4 ppm (tested using a validated PiCO+ Smokerlyzers (Bedfont^®^ Scientific Ltd., Kent, UK) were included since light smokers cover half of the overall smokers in Hong Kong [24]. Smokers that consented to participate in the QTW were invited to join a cluster RCT to evaluate the effects of two smoking cessation approaches delivered by brief advice (EC use was not part of the intervention): smoking reduction (gradually reduce consumption of conventional cigarettes to achieve abstinence in 3 months after enrollment) and quitting abruptly (stop using conventional cigarette as soon as possible) [30]. Those who did not join the RCT could still participate in the QTW.

The baseline questionnaire recorded participants’ socio-demographic characteristics, smoking behaviors, whether they had tried to abstain from conventional cigarettes (yes/no), and methods of quitting in their last cessation attempt before delivering assigned brief advice. Five telephone follow-ups (1 week, 1, 2, 3 and 6 months after baseline) were conducted and brief “booster” messages were delivered at the first 3 follow-ups. Number of cessation attempts and methods used to quit using conventional cigarettes since participation, smoking behaviors and self-reported past 7-day point prevalence abstinence (PPA) were recorded at all follow-ups. Ever EC use was first inquired at the 1-week follow-up because of time constraint at the on-site baseline survey and asked again at the 3-month follow-up, in which past 30-day EC use was additionally inquired. The QTW was approved by the Institutional Review Broad of the University of Hong Kong/Hospital Authority Hong Kong West Cluster (UW 14-382), and the present paper was based on a post hoc data analysis.

### 2.2. Measures

Ever and past 30-day EC use (yes/no) included even taking a single puff in respective periods. Participants reported ever EC use (at 1-week and 3-month) and past 30-day EC use (at 3-month). Those who reported ever EC use for the first time at the 3-month follow-up or past 30-day EC use at the same follow-up were analyzed together as EC users in the first 3 months of QTW. Those who reported abstinence from conventional cigarettes in the past 7 days (not even a single puff) [31] at 6 months were regarded as quitters. Quitters were invited for biochemical validation and abstinence from conventional cigarettes was confirmed by salivary cotinine <10 ng/mL using NicAlert test kit [32]. Heaviness of Smoking Index (HSI) [33] was computed by number of cigarettes per day (CPD; categorized into ≤10, 11–20, 21–30, >30) combined with time to first cigarette use after wake-up (within 5 min, 6–30 min, 31–60 min, >60 min) and HSI > 4 denoted a high level of nicotine dependence. Being abstinence for more than 24 h (yes/no) indicated a serious cessation attempt [34]. Participants also reported their planned abstinence date (within 7, 30, 60 days from baseline; not yet decided), and those who planned to quit within 30 days were classified as readier to quit, compared with those who planned to quit beyond 30 days or had no planned abstinence date, according to the Transtheoretical Model [35]. Smokers’ perceptions on the importance and confidence of successful abstinence from conventional cigarettes (a measure of their motivation to quit) were assessed on a scale of 0–10 [36]. Smokers’ perceptions on the difficulties of successful abstinence were assessed in a similar way [37].

### 2.3. Analysis

The current analyses included 956 participants who reported ever EC use status at the 1-week follow-up (92.8% of 1030 successfully followed smokers and 73.1% of all 1307 smokers in the QTW), regardless of the RCT participation (Table 1). Multiple logistic and linear regressions on complete cases yielded adjusted odds ratios (AOR) and raw coefficient b for quitting and smoking behaviors at the 6-month follow-up in relation to ever EC use at 1 week (referred to as “ever EC use” in the following unless otherwise specified) using Stata (Version 13.1: StataCorp LP, College Station, TX, USA), adjusting for lifetime cessation attempt, HSI, readiness to quit, CPD, perceptions on quitting and covariates measured at baseline when applicable. Similar analyses with missing observations treated as no cessation attempt nor cigarette reduction (intention to treat analyses, i.e., “missing = smoking”) and analyses based on propensity score (PS) stratification matching were conducted to test the robustness of results. PS was calculated by logistic regression using sex, age, cessation attempt, and HSI measured at baseline to predict ever EC use in R (Version 3.25: R Foundation for Statistical Computing, Vienna, Austria) [38,39]. Matched ever EC users and non-users would have similar characteristics except for ever EC use and therefore confounding variables would be effectively reduced before effect estimation. Supplementary analyses using EC use in the first 3 months after enrollment to predict abstinence from conventional cigarettes at 6 months (among 41 participants who were still smokers at the 3-month follow-up, another 7 users reported abstinence by then) were also performed. 

## 3. Results

### 3.1. Participants’ Baseline Characteristics

Participants being analyzed generally shared similar socio-demographic characteristics and smoking behaviors, with QTW smokers not included, except for higher CPD (15 vs. 13), of whom more had a high level of nicotine dependence (38.0% vs. 31.9%) and less had a monthly family income (US$1 = HK$7.8) < HK$20,000 (53.4% vs. 60.5%) (Data not shown in tables, all *p*s = 0.02). Our analytical samples (median age: 41; interquartile range, IQR: 30 to 54) were mostly male (81.8%), employed (76.8%), and had secondary education or above (83.9%), and more than half (53.3%) had a monthly household income <HK$20,000 (Table 2). They had a median CPD of 15 (IQR: 10 to 20) and 38% of them had a high level of nicotine dependence, 79.1% were ready to quit, 24.0% had never tried to abstain. Ever EC users (n = 163) had a higher median CPD, and more of them had a high level of nicotine dependence, while fewer had no previous cessation attempt, compared with non-users (all *p*s < 0.05). They also rated successful abstinence from conventional cigarettes as more important (*p* = 0.01) but held similar perceptions on confidence and difficulties of successful quitting.

### 3.2. Smoking and Quitting Behaviors at Follow-Up

The 3-month follow-up successfully interviewed 69.4% participants and the retention rates were similar between ever EC users (71.2%) and non-users (69.0%). The 6-month follow-up successfully interviewed 650 of 956 (67.9%) participants, including 111 of 163 (68.1%) ever EC users and 539 of 793 (68.0%) non-users. Among 650 successfully interviewed participants at the 6-month follow-up, 391 (60.2%) had made a cessation attempt, in which 299 reported the method they used to quit. A total of 122 (12.8%) reported abstinence from conventional cigarettes, comprised of 22 (19.8%) ever EC users and 100 (18.6%) non-users. Over half self-reported abstainers (73 of 122, 59.8%) quit without specific methods. Among 122 self-reported abstainers, 86 (Overall 70.5%, 81.8% in ever EC users vs. 68.0% in non-users) participated in the biochemical validation and 72 passed, including 16 (14.4%) ever EC users and 56 (10.4%) non-users. Regression analyses on complete cases showed ever EC use did not predict 7-day PPA (AOR 0.99, 95% CI 0.57 to 1.73), biochemically validated abstinence (AOR 1.22, 95% CI 0.64 to 2.34), making cessation attempt (AOR 0.74, 95% CI 0.48 to 1.14) nor smoking reduction by at least 50% (AOR 0.89, 95% CI 0.54 to 1.47), after adjusting for baseline cessation attempt, HSI, trial group and age and sex when applicable (Table 3). A similar pattern of estimates was observed for analyses under “missing = smoking” assumption. Among non-quitters at 6 months, ever EC use was not associated with readiness to quit (AOR 0.84, 95% CI 0.33 to 2.15) nor CPD (raw coefficient b 1.43, 95% CI −0.30 to 3.16) (Table 4) but appeared to be marginally significantly associated with perceived difficulties of abstinence (raw coefficient b 0.40, 95% CI −0.03 to 0.84). Estimations based on PS matching were similar to those from fully adjusted models, except for an insignificant higher cigarette reduction (raw coefficient b −1.62, 95% CI −5.89 to 2.65) and balance of characteristics after PS matching was checked to ensure robustness of the analyses.

Among 163 ever EC users at the beginning of QTW, 97 still smoked (47 lost to follow-up, 19 reported abstinence) at the 3-month follow-up, in which 7 smokers reported past 30-day EC use. Another 34 smokers newly reported ever EC use at the 3-month follow-up, forming 41 EC users during the first 3 months after enrollment and were still smoking at 3 months. Among these 41 EC users, EC use was also not associated with abstinence from conventional cigarettes at 6 months (AOR 1.02, 95% CI 0.22 to 4.71). At the 6-month follow-up, 22 of 163 initial ever EC users (89 smokers, 52 lost to follow-up) abstained from conventional cigarettes. These quitters had a lower median CPD (12.5 vs. 19), and more were ready to quit (100.0% vs. 74.5%) at baseline, compared with ever EC users who remained smoking (Both *p*s < 0.05) (Data not shown in tables). These quitters also perceived abstinence from conventional cigarettes as less difficult (5.9 vs. 7.6, *p* = 0.006) and had more confidence (7.4 vs. 5.1, *p* < 0.001) in it at baseline. 

## 4. Discussion

To our knowledge, this is the first report showing that ever EC use was not prospectively associated with abstinence from conventional cigarettes in Chinese adult smokers, most of whom were motivated to quit (as they were participants in a quitting contest), in a region where nicotine ECs are not widely available. Consistent null associations of ever EC use with biochemically validated abstinence, making cessation attempt or smoking reduction ≥50% were observed. The null associations of ever EC use with cigarette consumption amount, perceived difficulties on abstinence supported the robustness of our findings. Consistent non-significant findings were observed in analyses under “missing = smoking” assumption and based on PS. Longitudinal studies among smokers in the UK [40] and Italy [41] as well as among smokers seeking to quit in the US [42,43] also reported that EC use did not help smokers abstain.

Ever EC users who achieved abstinence at the 6-month follow-up had a low level of nicotine dependence and were readier to quit at baseline than those who had ever used EC but remained smoking, suggesting that ever EC use may have little effect on abstinence from conventional cigarette. This may be due to smokers were using ECs without nicotine, which are prevalent in HK [25] due to local legal restrictions. Nicotine-free EC may not effectively help a smoker abstain from conventional cigarettes since nicotine is a key to addiction to smoking and a prominent element in EC to be used for smoking cessation. Increased uptake of nicotine by greater intensity of EC use or using a liquid solution with higher nicotine strength would generally reduce smoking urge and boosted smoking abstinence more effectively [17,26,44]. More effective nicotine delivery by newer-generation ECs that have an improved delivery mechanism [21] enables them to outperform first-generation products on coping with withdrawal symptoms [45] and promoting abstinence [16], although the appearance and method of use of the newer generation products deviates from those of conventional cigarettes. EC without or with limited nicotine cannot satisfy smokers’ nicotine needs, but might only fulfill needs of non-nicotine factors (e.g., behavioral habit, psychosocial functions) in smoking [46] and therefore may have low efficacy as a smoking cessation aid. A recent report by the National Academies of Sciences, Engineering, and Medicine concluded that there is moderate evidence from RCTs to support the theory that ECs with nicotine are more effective than nicotine-free ECs for abstinence from conventional cigarettes [47]. However, this report also found insufficient evidence on effect comparisons for smoking cessation between EC and no treatment or to approved treatments [47]. It should also be noted that published studies focused on the first-generation ECs and no studies compared the newer generation ECs with proven cessation methods.

Another reason for the null effect of ever EC use on smoking abstinence might be experimental EC use for limited puffs/times or the last EC use occurred long before the commencement of our study given we included smokers who had ever used EC as low as a single puff. It is plausible that limited usage of EC would have minimal effect on quitting eventually, even if EC indeed promoted smoking cessation. We performed analysis in recent EC users (smokers who reported EC use during the first 3 months after enrollment) to partly address the EC experimentation problem and found a null association comparable to the result based on ever EC use at the start of QTW. It should also be noted that evidence from limited RCTs which suggested intensive EC use may promote quitting was inconclusive [15,26,48]. We also observed in 163 ever EC users at the beginning of QTW, only 7 of 97 smokers (47 lost to follow-up) had preceding 30-day EC use at the 3-month follow-up, suggesting many ever EC users may have quickly discontinued their trials on EC before they gave up smoking. EC experimental use for limited times was more commonly reported by those who first tried EC not for cessation in US adults [49], and we also found very few smokers (2.7%, 8 of 299) tried EC for quitting in our study. Consistently, most ever EC users (74.1%) did not perceive that EC might be helpful for smoking cessation at the beginning [29], although it was not clear whether previous experiences led to this perception.

Our results should be interpreted with caution. The current study included participants who had ever used EC as low as a single puff, and experimentation with EC by itself is unlikely to help smoking cessation. Detailed patterns of EC use, e.g., nicotine strength, consumption amount, use frequency, as well as product types, will affect nicotine delivery and subsequently abstinence from conventional cigarettes. Since EC was new in the market at that time and its users may have limited knowledge about it, such details were not investigated in our study, which limits the explanatory power of our findings. Future studies are urgently needed with a more in-depth investigation of EC product types and detailed use pattern. Biochemical validation was rejected by 29.5% quitters, mostly due to their busy schedule. Despite that, participation rate was higher among ever EC users which could only possibly bias the outcomes in favor of EC; we again found no significant association. The current observational study may be subject to selection bias, i.e., those who used EC and were able to abstain from conventional cigarettes are excluded from the study, although it is unlikely given a very low ever EC use prevalence in the overall adult population [25]. Our findings had a small sample size (e.g., 167 ever EC users at the beginning, 41 still smoking participants used EC in the first 3 months), which could limit the statistical power to detect a significant association if any. A post hoc power analysis shows that given 167 ever EC users and 793 non-users, assuming the true abstinence rate in non-user group is 13.5% we observed, we will be able to detect a true abstinence rate of 6.1% or 22.5% in ever EC users (α = 0.05, power = 0.8). The wide 95% confidence intervals of our results were also consistent with a small sample size and limited statistical power. Our results may have limited generalizability to other population since we were not able to rule out measured and unmeasured confounding variables, e.g., the included participants were only a subset of the QTW sample and they were more addicted to nicotine.

## 5. Conclusions

We found that ever use of EC, many of which were nicotine-free products, did not predict abstinence from conventional cigarettes among smokers who participated in the QTW contest in Hong Kong and was also not associated with indicators of future successful quitting. Studies with more detailed measurement on characteristics of EC use and a proper sample size are needed to confirm EC’s effect on quitting.

## Figures and Tables

**Table 1:** No. of smokers participated in the QTW Contest, RCT and included in this study.

Study Sample	n = 1307 (%)
Participated in the QTW Contest	1307 (100%)
Successfully interviewed at the 1-week follow-up	1030 (78.8%)
Answered questions on ever EC use	956 (73.1%)
	n = 956 (%)
Ever EC users	163 (17.1%)
Non-users	793 (82.9%)

QTW: “Quit to Win” contest. EC: electronic cigarette.

**Table 2:** Participants’ baseline characteristics.

	No EC Use (n = 793)	Ever EC Use (n = 163)	*p* Value ^a^
Male	82.7	77.3	0.10
Median age, years (IQR)	43 (32 to 55)	35 (28 to 47)	<0.001
Secondary education or above	84.1	92.5	0.01
In paid employment	75.8	81.7	0.11
Monthly household income <HK$20,000 (7.8 = 1 US$)	54.1	50.0	0.37
Median daily cigarette consumption (IQR)	15 (10 to 20)	19 (10 to 20)	<0.001
Heaviness of Smoking Index ≥4	36.6	44.8	0.05
No previous cessation attempt	25.5	16.6	0.02
Readier to quit	78.9	80.0	0.81
Perception on successful abstinence (mean ± SD) ^b^			
Importance	7.8 ± 2.2	8.3 ± 1.9	0.01
Confidence	5.8 ± 2.4	5.5 ± 2.5	0.17
Difficulties	7.2 ± 2.5	7.4 ± 2.7	0.50

IQR = Interquartile Range. ^a^ Wilcoxon rank-sum test for age and daily cigarette consumption. ^b^ Score 0–10, higher scores indicate higher levels of perception. EC: electronic cigarette. SD: standard deviation.

**Table 3:** Association of abstinence outcomes at the 6-month follow-up with ever EC use at 1-week follow-up.

Outcomes	Ever EC Use		Adjusted Odds Ratio (95% CI)		
	No (%)	Yes (%)	Model 1	Model 2 ^a^	Model 3 ^b^
Missing = no cessation attempt nor reduction (n = 956)					
Abstinence from conventional cigarettes	12.6	13.5	0.98 (0.59 to 1.62)	1.08 (0.64 to 1.83) ^c^	1.09 (0.65 to 1.85)
Biochemically validated quitting	7.1	9.8	1.28 (0.71 to 2.32)	1.34 (0.71 to 2.49) ^c^	1.33 (0.72 to 2.48)
Cessation attempt ^d^	40.9	41.1	0.96 (0.68 to 1.36)	0.92 (0.65 to 1.31)	0.95 (0.67 to 1.36)
Smoking reduction by at least 50%	19.6	17.2	0.91 (0.58 to 1.42)	0.92 (0.59 to 1.45)	0.89 (0.57 to 1.40)
Complete case analysis (n = 650)					
Abstinence from conventional cigarettes	18.6	19.8	0.93 (0.55 to 1.58)	0.99 (0.57 to 1.73) ^c^	1.04 (0.59 to 1.81)
Biochemically validated quitting	10.4	14.4	1.24 (0.67 to 2.29)	1.22 (0.64 to 2.34) ^c^	1.28 (0.67 to 2.44)
Cessation attempt ^d^	46.4	44.1	0.82 (0.54 to 1.25)	0.74 (0.48 to 1.14)	0.83 (0.54 to 1.28)
Smoking reduction by at least 50%	35.3	31.5	0.89 (0.54 to 1.46)	0.89 (0.54 to 1.47)	0.90 (0.55 to 1.49)

^a^ Model 2 adjusted for Model 1 variables and age, sex. ^b^ Model 3 is conditional logistic regression matched by propensity scores calculated from ever EC use on sex, age, baseline cessation attempt and Heaviness of Smoking Index, adjusted for group. ^c^ Adjusting for Model 2 variables and baseline cessation attempt and Heaviness of Smoking Index. ^d^ Adjusting for baseline cessation attempt in Model 1 and Model 2.

**Table 4:** Association of quitting and smoking outcomes at the 6-month follow-up with ever EC use at 1-week follow-up in non-quitters.

Outcomes	Ever EC Use (n = 528)		Unstandardized Coefficient b ^a^ (95% CI)	
	No (%)	Yes (%)	Model 1	Model 2 ^b^
Readier to quit ^c^	21.0	24.6	0.77 (0.31 to 1.94)	0.84 (0.33 to 2.15)
Median daily cigarette consumption (IQR)	10 (6 to 16)	10 (6 to 20)	1.40 (−0.32 to 3.12)	1.43 (−0.30 to 3.16)
Perception on successful abstinence (mean ± SD) ^d,e^				
Importance	7.4 ± 2.0	7.6 ± 1.9	0.14 (−0.36 to 0.64)	0.17 (−0.33 to 0.67)
Confidence	5.0 ± 2.2	4.8 ± 2.5	−0.05 (−0.56 to 0.47)	−0.06 (−0.58 to 0.46)
Difficulties	7.4 ± 1.9	7.8 ± 2.0	0.37 (−0.06 to 0.80)	0.40 (−0.03 to 0.84)

IQR = Interquartile Range. ^a^ Except adjusted odds ratio for readiness to quit. ^b^ Model 2 adjusted for Model 1 variables and age, sex. ^c^ Adjusting for baseline readiness to quit. ^d^ Score 0–10, higher scores indicate higher levels of perception. ^e^ Adjusting for baseline perceptions on quitting.

## References

[1] McNeill A., Brose L.S., Calder R., Hitchman S.C., Hajek P., McRobbie H. (2015). E-cigarettes: An Evidence Update. Public Health Engl..

[2] Schoenborn C.A., Gindi R.M. (2015). Electronic Cigarette Use among Adults: United States, 2014. NCHS Data Briefs.

[3] Lee J.A., Kim S.H., Cho H.-J. (2016). Electronic cigarette use among Korean adults. Int. J. Public Health.

[4] Farsalinos K.E., Poulas K., Voudris V., Le Houezec J. (2016). Electronic cigarette use in the European Union: Analysis of a representative sample of 27,460 Europeans from 28 countries. Addiction.

[5] Berg C.J., Haardoerfer R., Escoffery C., Zheng P., Kegler M. (2015). Cigarette Users’ Interest in Using or Switching to Electronic Nicotine Delivery Systems or Smokeless Tobacco for Harm Reduction, Cessation, or Novelty: A Cross-Sectional Survey of US Adults. Nicotine Tob. Res..

[6] Yao T., Jiang N., Grana R., Ling P.M., Glantz S.A. (2016). A content analysis of electronic cigarette manufacturer websites in China. Tob. Control.

[7] Pulvers K., Hayes R.B., Scheuermann T.S., Romero D.R., Emami A.S., Resnicow K., Olendzki E., Person S.D., Ahluwalia J.S. (2015). Tobacco Use, Quitting Behavior, and Health Characteristics Among Current Electronic Cigarette Users in a National Tri-Ethnic Adult Stable Smoker Sample. Nicotine Tob. Res..

[8] Hyland A. (2006). Individual-level predictors of cessation behaviours among participants in the International Tobacco Control (ITC) Four Country Survey. Tob. Control.

[9] Hartmann-Boyce J., McRobbie H., Bullen C., Begh R., Stead L.F., Hajek P. (2016). Electronic cigarettes for smoking cessation. Cochrane Database of Systematic Reviews.

[10] Rahman M.A., Hann N., Wilson A., Mnatzaganian G., Worrall-Carter L. (2015). E-Cigarettes and Smoking Cessation: Evidence from a Systematic Review and Meta-Analysis. PLoS ONE.

[11] Dib R.E., Suzumura E.A., Akl E.A., Gomaa H., Agarwal A., Chang Y., Prasad M., Ashoorion V., Heels-Ansdell D., Maziak W. (2017). Electronic nicotine delivery systems and/or electronic non-nicotine delivery systems for tobacco smoking cessation or reduction: A systematic review and meta-analysis. BMJ Open.

[12] Khoudigian S., Devji T., Lytvyn L., Campbell K., Hopkins R., O’Reilly D. (2016). The efficacy and short-term effects of electronic cigarettes as a method for smoking cessation: A systematic review and a meta-analysis. Int. J. Public Health.

[13] Kalkhoran S., Glantz S.A. (2016). E-cigarettes and smoking cessation in real-world and clinical settings: A systematic review and meta-analysis. Lancet Respir. Med..

[14] Adriaens K., Van Gucht D., Declerk P., Baeyens F. (2014). Effectiveness of the Electronic Cigarette: An Eight-Week Flemish Study with Six-Month Follow-up on Smoking Reduction, Craving and Experienced Benefits and Complaints. Int. J. Environ. Res. Public Health.

[15] Bullen C., Howe C., Laugesen M., McRobbie H., Parag V., Williman J., Walker N. (2013). Electronic cigarettes for smoking cessation: A randomised controlled trial. Lancet.

[16] Hitchman S.C., Brose L.S., Brown J., Robson D., McNeill A. (2015). Associations Between E-Cigarette Type, Frequency of Use, and Quitting Smoking: Findings from a Longitudinal Online Panel Survey in Great Britain. Nicotine Tob. Res..

[17] Biener L., Hargraves J.L. (2015). A Longitudinal Study of Electronic Cigarette Use among a Population-Based Sample of Adult Smokers: Association with Smoking Cessation and Motivation to Quit. Nicotine Tob. Res..

[18] Grana R.A., Popova L., Ling P.M. (2014). A Longitudinal Analysis of Electronic Cigarette Use and Smoking Cessation. JAMA Intern. Med..

[19] Al-Delaimy W.K., Myers M.G., Leas E.C., Strong D.R., Hofstetter C.R. (2015). E-Cigarette Use in the Past and Quitting Behavior in the Future: A Population-Based Study. Am. J. Public Health.

[20] Vansickel A.R., Eissenberg T. (2013). Electronic Cigarettes: Effective Nicotine Delivery after Acute Administration. Nicotine Tob. Res..

[21] Dawkins L., Kimber C., Puwanesarasa Y., Soar K. (2015). First- versus second-generation electronic cigarettes: Predictors of choice and effects on urge to smoke and withdrawal symptoms. Addiction.

[22] Kotz D., Brown J., West R. (2014). ‘Real-world’ effectiveness of smoking cessation treatments: A population study. Addiction.

[23] Ng M., Freeman M.K., Fleming T.D., Robinson M., Dwyer-Lindgren L., Thomson B., Wollum A., Sanman E., Wulf S., Lopez A.D. (2014). Smoking Prevalence and Cigarette Consumption in 187 Countries, 1980–2012. JAMA.

[24] (2016). Social Surveys Section (1) Thematic Household Survey Report No. 59.

[25] Cheung Y., Wang M., Ho S., Jiang N., Kwong A., Lai V., Lam T. (2017). Public Support for Electronic Cigarette Regulation in Hong Kong: A Population-Based Cross-Sectional Study. Int. J. Environ. Res. Public Health.

[26] Caponnetto P., Campagna D., Cibella F., Morjaria J.B., Caruso M., Russo C., Polosa R. (2013). Efficiency and Safety of an Electronic cigarette (ECLAT) as Tobacco Cigarettes Substitute: A Prospective 12-Month Randomized Control Design Study. PLoS ONE.

[27] Tseng T.-Y., Ostroff J.S., Campo A., Gerard M., Kirchner T., Rotrosen J., Shelley D. (2016). A Randomized Trial Comparing the Effect of Nicotine versus Placebo Electronic Cigarettes on Smoking Reduction among Young Adult Smokers. Nicotine Tob. Res..

[28] Meier E., Wahlquist A.E., Heckman B.W., Cummings K.M., Froeliger B., Carpenter M.J. (2016). A Pilot Randomized Crossover Trial of Electronic Cigarette Sampling Among Smokers. Nicotine Tob. Res..

[29] Wang M.P., Li W.H.C., Jiang N., Chu L.Y., Kwong A., Lai V., Lam T.H. (2015). E-Cigarette Awareness, Perceptions and Use among Community-Recruited Smokers in Hong Kong. PLoS ONE.

[30] Wang M.P., Li W.H., Cheung Y.T., Lam O.B., Wu Y., Kwong A.C., Lai V.W., Chan S.S., Lam T.H. (2017). Brief Advice on Smoking Reduction Versus Abrupt Quitting for Smoking Cessation in Chinese Smokers: A Cluster Randomized Controlled Trial. Nicotine Tob. Res..

[31] West R., Hajek P., Stead L., Stapleton J. (2005). Outcome criteria in smoking cessation trials: Proposal for a common standard. Addiction.

[32] Cooke F., Bullen C., Whittaker R., McRobbie H., Chen M.-H., Walker N. (2008). Diagnostic accuracy of NicAlert cotinine test strips in saliva for verifying smoking status. Nicotine Tob. Res..

[33] Heatherton T.F., Kozlowski L.T., Frecker R.C., Rickert W., Robinson J. (1989). Measuring the Heaviness of Smoking: Using self-reported time to the first cigarette of the day and number of cigarettes smoked per day. Br. J. Addict..

[34] Carpenter M.J., Hughes J.R. (2005). Defining Quit Attempts: What Difference Does a Day Make?. Addiction.

[35] Prochaska J.O., Velicer W.F. (1997). The transtheoretical model of health behavior change. Am. J. Health Promot..

[36] Rollnick S., Miller W.R., Heather N. (1998). Readiness, importance, and confidence: Critical conditions of change in treatment. Treating Addictive Behaviors.

[37] Lam T.-H., Abdullah A.S.M., Chan S.S.C., Hedley A.J., Hong Kong Council on Smoking and Health Smoking Cessation Health Centre (SCHC) Steering Group (2005). Adherence to nicotine replacement therapy versus quitting smoking among Chinese smokers: A preliminary investigation. Psychopharmacology.

[38] Rosenbaum P.R., Rubin D.B. (1983). The central role of the propensity score in observational studies for causal effects. Biometrika.

[39] Caliendo M., Kopeinig S. (2008). Some Practical Guidance for the Implementation of Propensity Score Matching. J. Econ. Surv..

[40] Brose L.S., Hitchman S.C., Brown J., West R., McNeill A. (2015). Is the use of electronic cigarettes while smoking associated with smoking cessation attempts, cessation and reduced cigarette consumption? A survey with a 1-year follow-up: E-cigarettes, cessation, attempts, reduction. Addiction.

[41] Manzoli L., Flacco M.E., Fiore M., Vecchia C.L., Marzuillo C., Gualano M.R., Liguori G., Cicolini G., Capasso L., D’Amario C. (2015). Electronic Cigarettes Efficacy and Safety at 12 Months: Cohort Study. PLoS ONE.

[42] Pearson J.L., Stanton C.A., Cha S., Niaura R.S., Luta G., Graham A.L. (2015). E-Cigarettes and Smoking Cessation: Insights and Cautions from a Secondary Analysis of Data from a Study of Online Treatment-Seeking Smokers. Nicotine Tob. Res..

[43] Vickerman K.A., Carpenter K.M., Altman T., Nash C.M., Zbikowski S.M. (2013). Use of Electronic Cigarettes among State Tobacco Cessation Quitline Callers. Nicotine Tob. Res..

[44] Lechner W.V., Tackett A.P., Grant D.M., Tahirkheli N.N., Driskill L.M., Wagener T.L. (2015). Effects of Duration of Electronic Cigarette Use. Nicotine Tob. Res..

[45] Lechner W.V., Meier E., Wiener J.L., Grant D.M., Gilmore J., Judah M.R., Mills A.C., Wagener T.L. (2015). The comparative efficacy of first- versus second-generation electronic cigarettes in reducing symptoms of nicotine withdrawal. Addiction.

[46] Fagerström K. (2012). Determinants of Tobacco Use and Renaming the FTND to the Fagerström Test for Cigarette Dependence. Nicotine Tob. Res..

[47] National Academies of Sciences, Engineering, and Medicine (2018). Public Health Consequences of E-Cigarettes.

[48] Malas M., van der Tempel J., Schwartz R., Minichiello A., Lightfoot C., Noormohamed A., Andrews J., Zawertailo L., Ferrence R. (2016). Electronic Cigarettes for Smoking Cessation: A Systematic Review. Nicotine Tob. Res..

[49] Pepper J.K., Ribisl K.M., Emery S.L., Brewer N.T. (2014). Reasons for Starting and Stopping Electronic Cigarette Use. Int. J. Environ. Res. Public Health.

